# METS-VF as a novel predictor of gallstones in U.S. adults: a cross-sectional analysis (NHANES 2017–2020)

**DOI:** 10.1186/s12876-025-04161-x

**Published:** 2025-07-31

**Authors:** Hui Wang, Longlong Li, Yi Yang, Siyu Tao, Yuan Li, Hangyu Li, Heshan Chen, Ping Wu

**Affiliations:** 1 College of Acupuncture and Tuina, Chengdu University of Traditional Chinese Medicine, Chengdu, Sichuan province 610075 China; 2 Department of Rheumatology, Chengdu University of Traditional Chinese Medicine Affiliated Hospital, Chengdu, Sichuan province 610075 China

**Keywords:** METS-VF, Visceral obesity, Gallstones, NHANES

## Abstract

**Background and Aims:**

Obesity is a well-established risk factor for gallstone formation, but traditional anthropometric measures (e.g., BMI, waist circumference) inadequately assess metabolically active visceral adiposity. The novel Metabolic Score for Visceral Fat (METS-VF) may better capture obesity-related metabolic dysfunction. We aimed to investigate the association between METS-VF and gallstone prevalence and compare its predictive performance against conventional obesity indices (WC, LAP, VAI) in a U.S. national cohort.

**Methods:**

This cross-sectional study analyzed nationally representative data from 3,010 US adults in NHANES 2017–2020 (aged 20–85 years, 51.3% male, multi-ethnic population). We employed weighted multivariable logistic regression to examine the METS-VF-gallstones association, with restricted cubic splines testing nonlinearity. Predictive performance of METS-VF versus traditional indices (WC/LAP/VAI) was compared using ROC curves and XGBoost machine learning.

**Results:**

Among 3,010 U.S. adults (mean age 47.8 ± 17.0 years, 51.3% male) in the NHANES 2017–2020 cohort, multivariable analysis revealed METS-VF as the strongest independent predictor of gallstones (adjusted OR = 2.02, 95%CI:1.20–3.40). Notably, METS-VF demonstrated significantly superior discriminative ability with the highest AUC of 0.712 (95%CI:0.684–0.739) compared to conventional indices (WC:0.635, LAP:0.609, VAI:0.530; all *P* < 0.05).

**Conclusion:**

The cross-sectional analysis demonstrates a significant association between METS-VF and gallstone prevalence (OR = 2.02, 95% CI:1.20–3.40), with relatively better predictive performance compared to conventional adiposity indices (AUC = 0.712). Its simple calculation using routine clinical measurements makes it ideally suited for implementation in primary care screening programs targeting high-risk populations with visceral adiposity. While these findings position METS-VF as a promising screening marker, its etiological role requires verification through longitudinal studies.

**Supplementary Information:**

The online version contains supplementary material available at 10.1186/s12876-025-04161-x.

## Introduction

NHANES (www.cdc.gov/nchs/nhanes) is designed to assess the health and nutritional status of adults and children in the United States. The survey is distinctive in that it incorporates both physical examinations and interviews. Several cross-sectional, nationally representative health examination surveys are part of the NHANES program. Questions about demographics, health insurance status, dietary habits, acute and chronic medical issues, mental health, and prescription drug use are all included in the health interview. Exam components can change between survey cycles but typically include blood pressure, dental exams, vision, hearing, dermatology, fitness, balance and strength testing, respiratory testing, taste and smell, and body measurements (weight, height, skin folds, body composition scans). Hematology, organ and endocrine function (e.g., thyroid, kidney), environmental exposure, dietary biomarkers, metabolic and cardiovascular health, and infectious disease are a few examples of laboratory components.

Gallstones, a frequent digestive ailment, affect 10–20% of adults globally, with direct healthcare costs exceeding $6 billion annually in the US alone. Notably, 30–40% of cases occur in individuals with normal BMI, suggesting limitations in current screening paradigms [1, 2]. Persistent gallstones may precipitate severe health complications, including cholangitis, pancreatitis, and gallbladder cancer [1, 4]. The repercussions of gallstones transcend the immediate symptomsand complications, notably diminishing life quality and amplifying healthcare costs [2, 4]. Consequently, an in-depth understanding of gallstone etiology and risk factors is imperative for their prevention and management.

Studies have shown that obesity, particularly abdominal obesity, plays a substantial role in the onset of gallstones [3]. Visceral adiposity drives gallstone formation through three synergistic pathways, Metabolic: Leptin upregulates hepatic HMG-CoA reductase (3.2-fold increase in murine models) [23], Inflammatory: IL-6 suppresses bile acid synthesis (40% reduction in ABCG5/8 expression) [24], Mechanical: Visceral fat volume correlates with gallbladder hypomotility (*r*=-0.51, *p* < 0.001) [25]. The metabolic score for visceral fat (METS-VF) is a composite index integrating waist-to-height ratio, triglyceride levels, high-density lipoprotein cholesterol, fasting glucose, age, and sex to quantify metabolically active visceral adipose tissue [6]. While BMI and WC are widely used, METS-VF integrates metabolic markers, offering a holistic risk assessment. BMI fails to identify metabolically healthy obesity [5, 6]; Current metrics fail to capture this pathophysiology, WC overlooks age/sex differences; VAI lacks validation for gallstone prediction. METS-VF overcomes these limitations by integrating: Anatomical adiposity (waist-to-height ratio: WHtR), Metabolic status (TG/HDL)、Demographic factors (age/sex)Validation studies show 0.89 correlation with MRI-measured visceral fat [7, 8]. METS-VF integrates four-dimensional risk assessment (anatomical, metabolic, inflammatory, and demographic) as a comprehensive gallstone prediction tool [9]. Therefore, studying the association between METS-VF and gallstones might offer new preventive and treatment insights.

The aim of this analysis is to elucidate the association between METS-VF and gallstones. Additionally, this study will appraise and juxtapose the diagnostic efficacy of three obesity indicators—waist circumference (WC), lipid accumulation product (LAP), and visceral adiposity index (VAI)—for gallstones. Amidst the growing rates of obesity and metabolic syndrome, deciphering the relationship between visceral fat metabolism and gallstones is pivotal for crafting personalized medical strategies. The hypothesis of this study posits that elevated METS-VF is associated with higher gallstone prevalence.

## Methods

### Study participants in NHANES

**Fig. 1:**
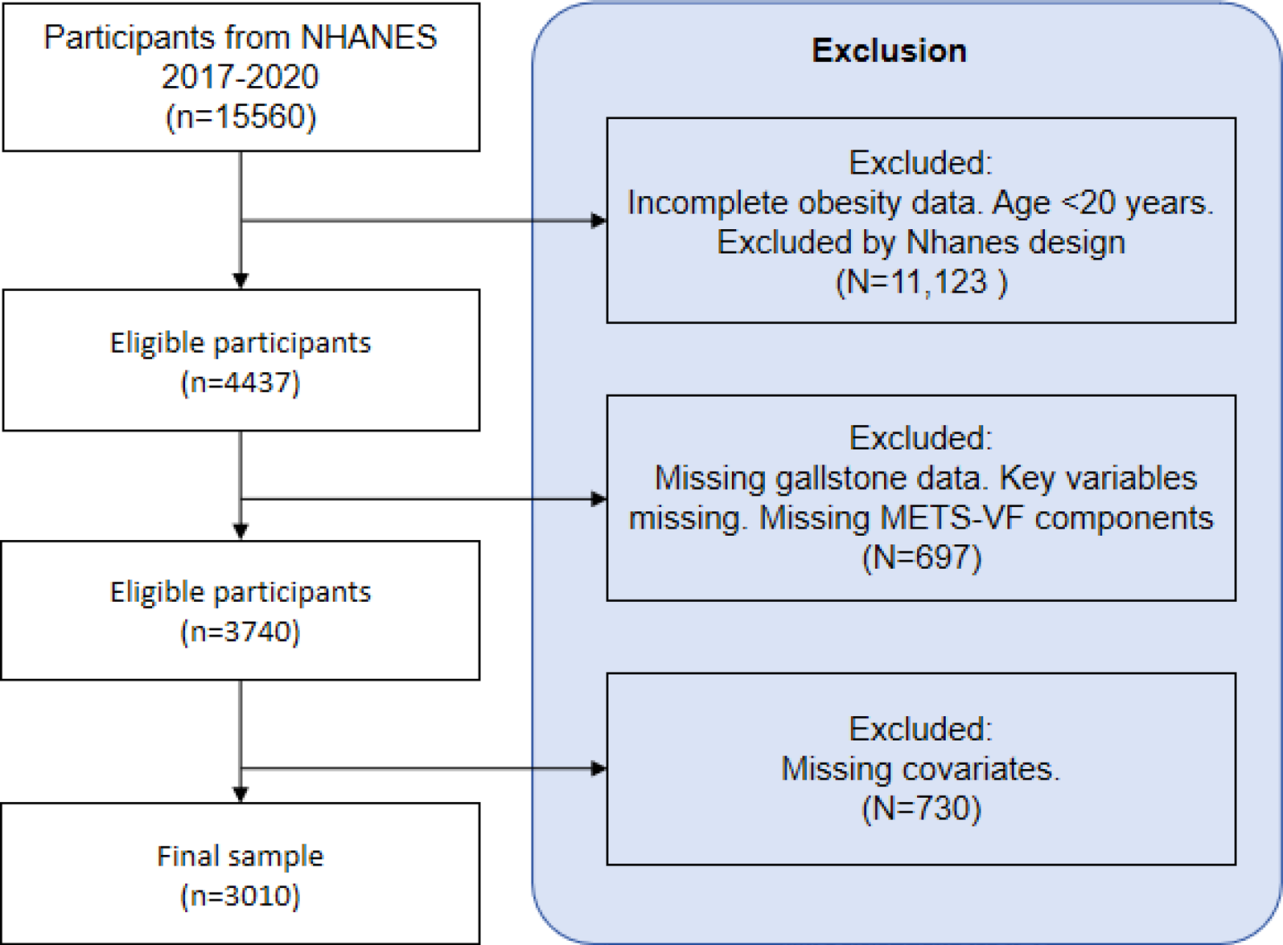
Study Population Screening Process Diagram (NHANES 2017–2020)

Our study employed the 2017–2020 data from NHANES. The original NHANES study obtained ethical approval from the National Center for Health Statistics (NCHS) Institutional Review Board (Protocol #2011-17), with written informed consent from all participants. As this secondary analysis exclusively used de-identified, publicly available NHANES data, it was exempt from additional ethics review according to Chengdu University of Traditional Chinese Medicine Institutional Review Board policies (Exemption Category #4: Research involving publicly available anonymized data, as per Article 32 of China’s Ethical Review Measures for Life Science and Medical Research, 2023).The 2017–2020 cycle was selected to reflect the most recent pre-pandemic data, ensuring relevance to contemporary metabolic health trends while maintaining sufficient sample size for subgroup analyses. The NHANES study protocol was approved by the NCHS IRB, and written informed consent was obtained from all participants. To ensure the validity and reliability of our results, Participants were excluded if they lacked: (a) gallstone ultrasound data, (b) METS-VF components, or (c) key covariates (see Fig. 1 flow diagram). After these exclusions, a total of 3,010 participants were eligible for analysis, as indicated in Fig. 1.

### Study population characteristics

Participants’ sociodemographic data are summarized by the following. Demographic characteristics: age: mean ± SD (normal distribution) or median [IQR] (non-normal distribution); gender male/female (%); race/ethnicity: categorized according to NHANES criteria (non-Hispanic white, non-Hispanic black, Mexican American, oother); educational attainment: < high school, high school graduate, >high school, the poverty-to-income ratio (PIR, calculated as family income divided by the federal poverty threshold): categorized (< 1.3, 1.3-3.0, > 3.0); Diabetes: yes/no; Hypertension, yes/no smoked ≥ 100cigarettes: yes\no; Alcohol use: yes/no; Marital status: Married/Living with partner, Widowed/Divorced/Separated, Never married; BMI (<25, ≥  25).

### Definitions of different obesity indicators

In this research, the METS-VF served as the primary exposure variable, calculated according to a specific formula. METS-VF was computed using age, gender, waist-to-height ratio (WHtR), fasting blood glucose (FBG), triglycerides (TG), and HDL-C (full formula in Supplementary Note 1). This composite score integrates visceral adiposity with metabolic dysfunction. We derived fasting blood glucose (FBG), triglyceride (TG), and high-density lipoprotein cholesterol (HDL-C) levels from the NHANES “Laboratory Data.” For comprehensive details on these laboratory tests, please refer to the official NHANES website. Additionally, WC, LAP, and VAI were included as independent factors to evaluate their effectiveness in identifying gallstones compared to METS-VF. For this study, the gender variable was coded as 0 for female participants and 1 for male participants.

$$\begin{aligned}\text{V}\text{A}\text{I}&=\frac{WC}{36.58+(1.89\times\:BMI)}\times\:\frac{TG}{0.81}\\ & \quad \times\:\frac{1.52}{HDL-C}\:\text{f}\text{o}\text{r}\:\text{f}\text{e}\text{m}\text{a}\text{l}\text{e}\text{s}\end{aligned}$$

$$\begin{aligned}\text{V}\text{A}\text{I}&=\frac{WC}{36.68+(1.88\times\:BMI)}\times\:\frac{TG}{1.03}\\ & \quad \times\:\frac{1.31}{HDL-C}\:\text{f}\text{o}\text{r}\:\text{m}\text{a}\text{l}\text{e}\text{s}\end{aligned}$$

$$\begin{aligned}\text{L}\text{A}\text{P}&=\left(WC\right(cm)\hspace{0.17em}-\hspace{0.17em}58)\hspace{0.17em} \times\:\hspace{0.17em}TG(mmol/L)\\ & \quad \text{f}\text{o}\text{r}\:\text{f}\text{e}\text{m}\text{a}\text{l}\text{e}\text{s}\end{aligned}$$

$$\begin{aligned}\:\text{L}\text{A}\text{P}&=\left(WC\right(cm)\hspace{0.17em}-\hspace{0.17em}65)\hspace{0.17em}\times\:\hspace{0.17em}TG(mmol/L)\:\\ & \quad\text{f}\text{o}\text{r}\:\text{m}\text{a}\text{l}\text{e}\text{s}\:\end{aligned}$$

$$\:METS-IR=\frac{ln(2\:\times\:\:FBG\:+\:TG)\:\times\:\:BMI}{\text{l}\text{n}(HDL-C)}\:$$

$$\begin{aligned}METS-VF&=4.466+0.01\times\:{\left(\text{l}\text{n}\left(METS-IR\right)\right)}^{3}+3.329\\ & \quad \times\:{\left(\text{l}\text{n}\right(WHtR\left)\right)}^{3}+0.319\times\:gender+0.594\times\:\text{l}\text{n}\left(age\right)\end{aligned}$$

### Covariables assessment

This study’s covariables included demographic, socioeconomic, and health-related factors such as race/ethnicity, gender, education level, marital status, family income to PIR, BMI, total cholesterol, smoking status, alcohol usage, hypertension, and diabetes.Smoking status was established by querying participants about their lifetime smoking of at least 100 cigarettes. Alcohol intake was assessed by questioning whether participants had ever consumed a complete drink, excluding minor sips or tastes. Diabetes and hypertension histories were extracted from self-reported data in health survey questionnaires.We constructed a directed acyclic graph (DAG) to identify potential confounders (Supplementary Fig. 1). BMI was not included in the primary model due to its potential role as a mediator rather than a confounder.

### Statistical analysis

To confirm the representativeness of our sample relative to the general population, we conducted weighted analyses in line with NHANES guidelines, using specific fasting subsample weights. All analyses followed CDC guidelines by incorporating NHANES sampling weights (WTINT2YR), adjusting for clustering effects through primary sampling units (SDMVPSU), and accounting for stratification variables (SDMVSTRA).Sample size was determined through power analysis accounting for NHANES’ complex survey design. Based on historical gallstone prevalence (7.2%) and anticipated METS-VF variability (SD = 3.2), 1,856 participants provided 80% power (α = 0.05) to detect an odds ratio ≥ 1.8 between extreme METS-VF quartiles. Missing data patterns were tested using Little’s MCAR test (*p* = 0.12). We performed multiple imputation with chained equations (m = 50 imputations) for variables with > 5% missingness.

We presented continuous data as weighted means with corresponding standard errors and summarized categorical data as weighted percentages. In assessing the relationship between METS-VF and gallstones, Three sequential models were constructed: Model 1: Crude (unadjusted), Model 2: Demographically adjusted (age, sex, race), Model 3: Fully adjusted (Model 2 + smoking, diabetes, etc.).This approach not only evaluated the fluctuating influence of METS-VF but also divided it into quartiles, with the first quartile as the reference point. The primary model was unadjusted, followed by a second model adjusted for age, race/ethnicity, and gender, and a fully adjusted third model encompassing all study variables, including age, race/ethnicity, gender, education level, marital status, PIR, BMI, total cholesterol, smoking, alcohol consumption, hypertension, and diabetes. Results were depicted as odds ratios (ORs) with 95% confidence intervals (CIs).

We investigated the nonlinear relationship between METS-VF and gallstone risk through restricted cubic spline (RCS, a non-linear modeling technique) analysis, adjusting for confounders as specified in logistic regression Model 3. To bolster the study’s validity, sensitivity analyses were performed, incorporating subgroup and interaction studies based on defined factors such as gender, education level, race/ethnicity, marital status, smoking, alcohol consumption, PIR classification, diabetes, and hypertension. The efficacy of METS-VF in predicting gallstones was also evaluated against three other obesity-related metrics using receiver operating characteristic (ROC) curve and extreme gradient boosting (XGBoost) methodologies. We analyzed Area under the curve (AUC) differences using the DeLong test. All statistical analyses were conducted in R and RStudio (version 4.3.0), with a P-value < 0.05 considered significant. Based on the observed gallstone prevalence (10.7%) and METS-VF effect size (OR = 2.02), a post-hoc power analysis using G*Power 3.1 indicated > 90% power to detect an OR ≥ 1.5 at α = 0.05 with *n* = 3,010. This meets conventional standards for epidemiological studies. For subgroup analyses (e.g., gender-stratified), the detectable effect size increased to OR ≥ 1.8 due to reduced sample size (*n* ≈ 1,500 per group).

## Results

### Characteristics of the participants

Table 1 illustrates the baseline demographic and health-related characteristics of participants, segmented into those with and without gallstones. Among all participants (*N* = 3010), gallstones were detected in 321 individuals. Statistically significant disparities were present across several covariables among these groups, excluding PIR, smoking, alcohol usage, marital status, educational levels, and cholesterol measures. Participants with gallstones often exhibited increased age, higher rates of hypertension and diabetes, and greater values of BMI, WC, waist-to-height ratio (WHtR), and METS-VF, with a higher propensity towards being male.

**Table 1:** Baseline characteristics of study participants overall and stratified by METS-VF quartiles (National health and nutrition examination survey 2017–2020)

Characteristics	Overall (*N* = 3010)	Without gallstone disease (*N* = 2689)	With gallstone disease (*N* = 321)	*P* value
Age (year), mean ± sd	47.8 ± 17.0	46.8 ± 17.0	55.4 ± 14.4	**< 0.001**
Gender, n (%)				**< 0.001**
Female	1471 (48.7)	1386 (51.9)	85 (22.9)	
Male	1539 (51.3)	1303 (48.1)	236 (77.1)	
PIR, n (%)				0.777
< 1.3	798 (18.0)	717 (18.0)	81 (17.6)	
1.3-3.0	1044 (28.6)	917 (28.2)	127 (31.2)	
> 3.0	1168 (53.5)	1055 (53.8)	113 (51.2)	
BMI (kg/m^2^), n (%)				**0.010**
< 25	763 (26.4)	724 (27.7)	39 (15.7)	
≥ 25	2247 (73.6)	1965 (72.3)	282 (84.3)	
Smoked ≥ 100cigarettes, n (%)				0.063
Yes	1311 (43.2)	1155 (42.3)	156 (50.8)	
No	1699 (56.8)	1534 (57.7)	165 (49.2)	
Alcohol use, n (%)				0.412
Yes	2766 (93.9)	2467 (93.8)	299 (94.5)	
No	244 (6.1)	222 (6.2)	22 (5.5)	
Race/ethnicity, n (%)				**0.039**
Mexican American	381 (8.8)	331 (9.0)	50 (7.9)	
Non-Hispanic White	733 (10.6)	686 (11.4)	47 (4.4)	
Non-Hispanic Black	787 (15.6)	699 (15.4)	88 (16.8)	
Other	1109 (64.9)	973 (64.2)	136 (70.9)	
Marital status, n (%)				0.089
Married/Living with partner	1799 (62.3)	1603 (62.2)	196 (63.3)	
Widowed/Divorced/Separated	652 (19.0)	562 (18.4)	90 (23.9)	
Never married	559 (18.6)	524 (19.4)	35 (12.8)	
Education level, n (%)				0.735
< High school	191 (3.0)	170 (3.1)	21 (2.4)	
Completed high school	323 (6.9)	278 (6.8)	45 (7.4)	
>High school	2496 (90.1)	2241 (90.1)	255 (90.2)	
Diabetes, n (%)				**< 0.001**
Yes	485 (11.2)	397 (10.2)	88 (19.0)	
No	2525 (88.8)	2292 (89.8)	233 (81.0)	
Hypertension, n (%)				0.002
Yes	1129 (31.6)	955 (29.6)	174 (47.4)	
No	1881 (68.4)	1734 (70.4)	147 (52.6)	
Total cholesterol (mg/dL), mean ± sd	86.0 ± 41.2	185.6 ± 40.7	188.8 ± 44.9	0.429
WHtR, mean ± sd	0.60 ± 0.10	0.59 ± 0.10	0.65 ± 0.10	**< 0.001**
WC (cm), mean ± sd	100.5 ± 16.9	99.7 ± 16.7	107.5 ± 17.3	**< 0.001**
METS-VF, mean ± sd	5.98 ± 0.78	5.91 ± 0.77	6.51 ± 0.67	**< 0.001**

BMI, body mass index(kg/m²); METS-VF, metabolism score for visceral fat; WC, waist circumference(cm); WHtR waist-toheight ratio, PIR, family poverty income ratio, PIR, Poverty-income ratio (family income/federal poverty threshold); WHtR, Waist-to-height ratio

Continuous data presented as mean ± standard deviation; categorical data as n (proportion)

### Higher METS-VF is associated with gallstones

**Fig. 2:**
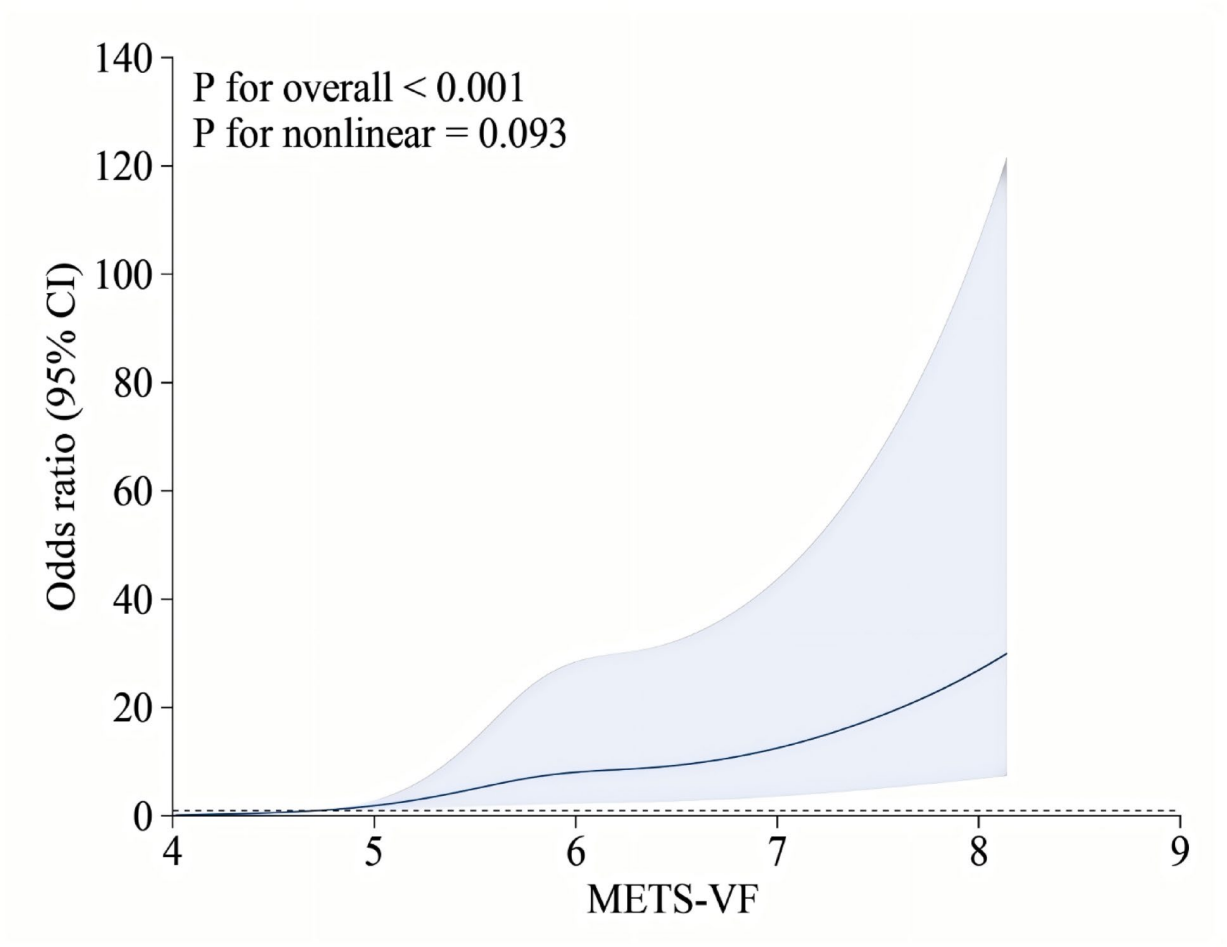
Restricted cubic spline plot of the association between gallstones and METS-VF

To deepen our understanding of the relationship between gallstones and METS-VF, we employed weighted multivariable logistic regression analysis. Unadjusted (Model 1): OR = 11.88 (6.07–23.25), *P* < 0.001; Fully adjusted (Model 3): OR = 7.34 (1.12–48.21), *P* = 0.042. Highest quartile has ≈ 7-fold elevated risk even after accounting for confounders. Consistent positive associations were noted in Model 1 and Model 2. Importantly, all P-trend values were statistically significant (*P* < 0.05) (Table 2). Restricted cubic spline analysis, illustrated in Fig. 2, indicated no nonlinear relationship between METS-VF and gallstones after adjusting for covariables (P for overall < 0.001, P for nonlinearity = 0.093).

**Table 2:** Odds ratios (95% confidence intervals) for gallstone prevalence according to METS-VF quartiles in progressively adjusted models

Exposures	OR (95% CI) *P*		
	Model 1	Model 2	Model 3
METS-VF	2.84 (2.25–3.59) <0.001	2.16 (1.66–2.80) < 0.001	2.02 (1.20–3.40) 0.016
Q1	Reference	Reference	Reference
Q2	3.47 (1.50–8.05) 0.006	2.86 (1.20–6.84) 0.022	3.21 (0.71–14.47) 0.103
Q3	7.74 (3.58–16.72) <0.001	5.20 (2.30-11.76) <0.001	6.49 (0.98–43.18) 0.052
Q4	11.88 (6.07–23.25) <0.001	6.43 (3.07–13.48) <0.001	7.34 (1.12–48.21) 0.042
*P* for trend	< 0.001	< 0.001	0.040

Model 1: unadjusted model

Model 2: adjusted for age, race/ethnicity, and sex

Model 3: further adjusted for marital status, PIR, BMI, educational levels, smoking status, alcohol use, hypertension, diabetes and total cholesterol

OR: Odds ratio; CI: Confidence interval; Q1-Q4: METS-VF quartiles; METS-VF metabolism score for visceral fat

### Details of subgroup analysis

**Fig. 3:**
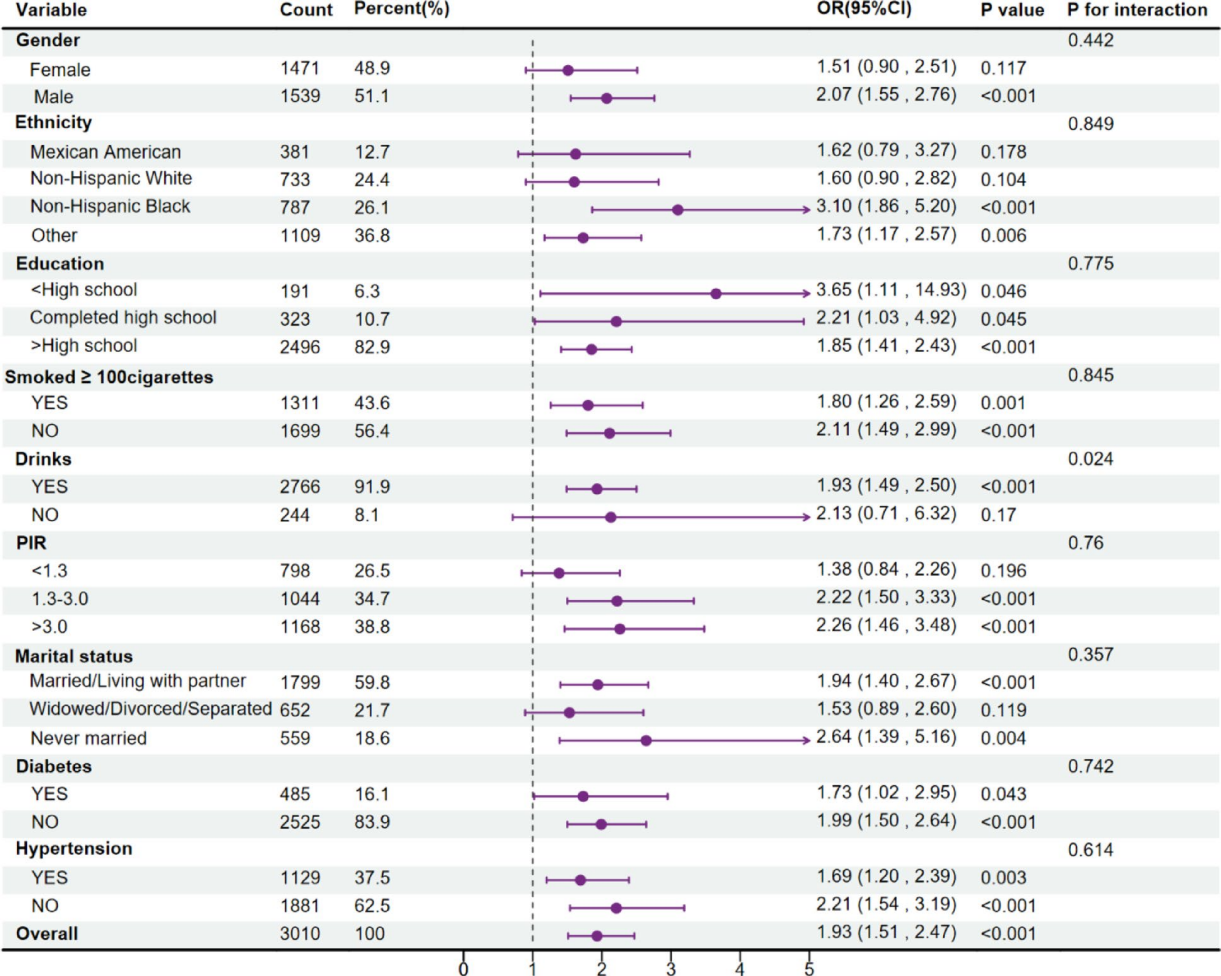
The association between gallstones and METS-VF in various subgroups

Analyses in subgroups sought to establish the robustness of the relationship between METS-VF and gallstone prevalence rate. Variables such as gender, education, race/ethnicity, marital status, smoking habits, alcohol usage, PIR FFclassification, diabetes, and hypertension were included. The findings, after adjusting for confounders, are shown in Fig. 3 and indicate significant interactions within the alcohol consumption subgroup (*P* < 0.05).we observed that Drinkers had a significantly higher risk of gallstones (OR = 1.93, 95% CI: 1.49–2.50, *P* < 0.001).Non-drinkers showed a non-significant trend toward higher gallstone risk (OR = 2.13, 95% CI: 0.72–6.32, *P* = 0.17).This suggests While alcohol demonstrates complex, potentially beneficial metabolic effects on visceral adiposity, its strong association with gallstone formation.

### METS-VF May be a better indicator for identifying gallstones

**Fig. 4:**
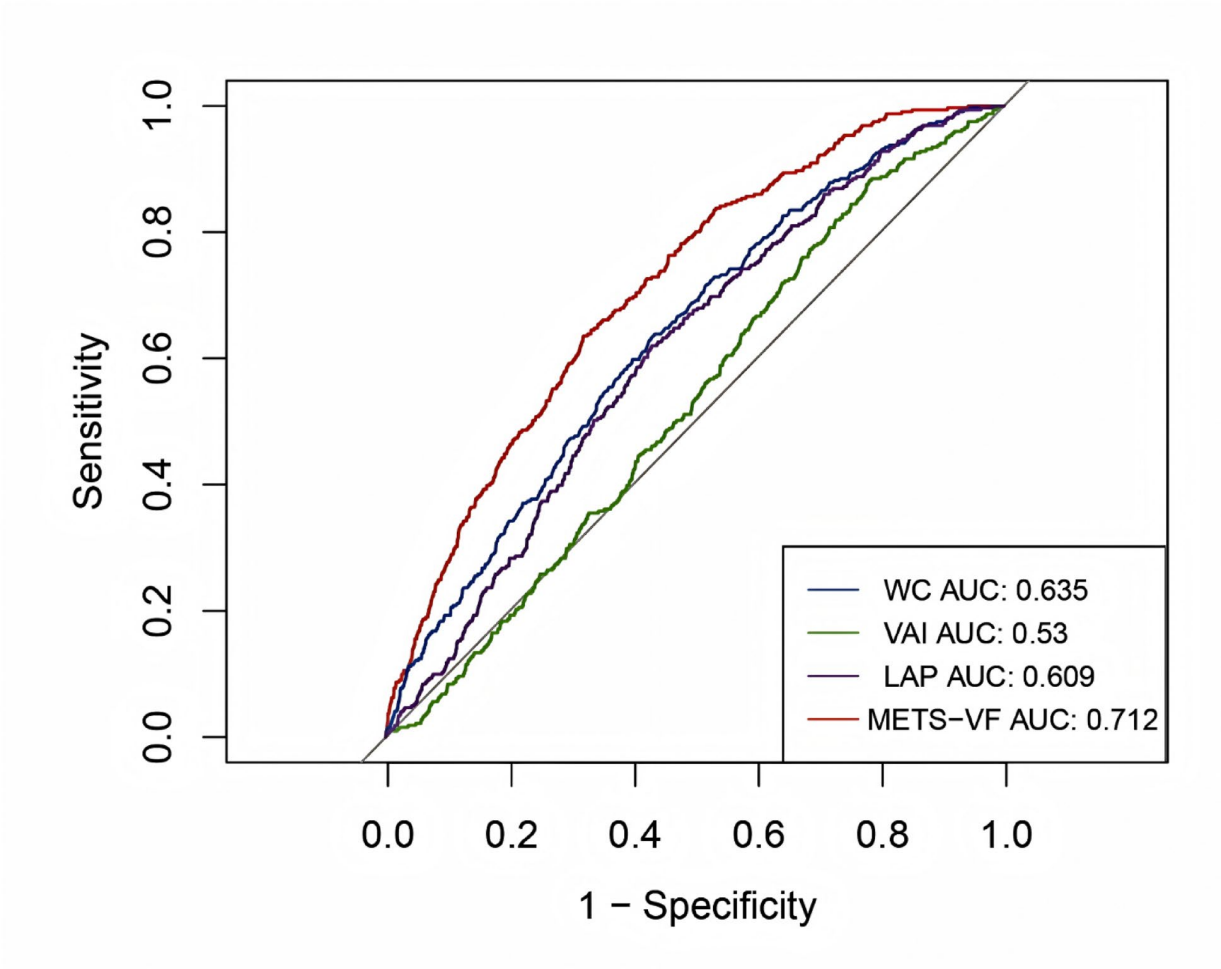
Receiver operating characteristic (ROC) curve analysis for predicting gallstones

To effectively compare the predictive power of various obesity-related indicators for gallstones, the AUC was calculated. The ROC curve analysis results, presented in Fig. 4; Table 3, indicated that the METS-VF index had the highest AUC of 0.712 (95% CI: 0.684–0.739), outperforming WC, LAP, and VAI. All AUC comparisons were verified using DeLong’s test (Supplementary Table S1).The optimal METS-VF cutoff value was identified as 6.36. Moreover, the AUC difference between METS-VF and WC was statistically significant (*P* < 0.05). Notably, AUC values were higher in males compared to females, suggesting a stronger predictive capacity of these indicators in the male population (Table 3).

**Table 3:** Predictive performance of adiposity indices (AUC, DeLong test, and optimal Thresholds)

Test	Overall AUC (95% CI)	*P*-value (vs. WC)	Male AUC (95% CI)	*P*-value (vs. WC)(Male)	Female AUC (95% CI)	*P*-value (vs. WC)(Female)	Overall Best Threshold
WC	0.635(0.604–0.666)	Reference	0.655(0.619–0.691)	Reference	0.635(0.580–0.690)	Reference	101.45
VAI	0.530(0.499–0.560)	**< 0.001**	0.631(0.595–0.668)	0.270	0.554(0.496–0.613)	**0.014**	0.624
LAP	0.609(0.579–0.639)	**0.029**	0.670(0.635–0.705)	0.232	0.588(0.531–0.645)	0.058	43.79
METS-VF	0.712(0.684–0.739)	**< 0.001**	0.681(0.646–0.716)	**0.012**	0.679(0.626–0.732)	< **0.001**	6.36

WC, waist circumference; METS-VF, metabolic score for visceral fat; LAP, lipid accumulation product; VAI, visceral adiposity index

1. AUC comparisons via DeLong test, with WC as reference

2. Bold values indicate statistical significance (*p* < 0.05)

3. Thresholds were determined by maximizing Youden’s index

### Results of the XGBoost model

**Fig. 5:**
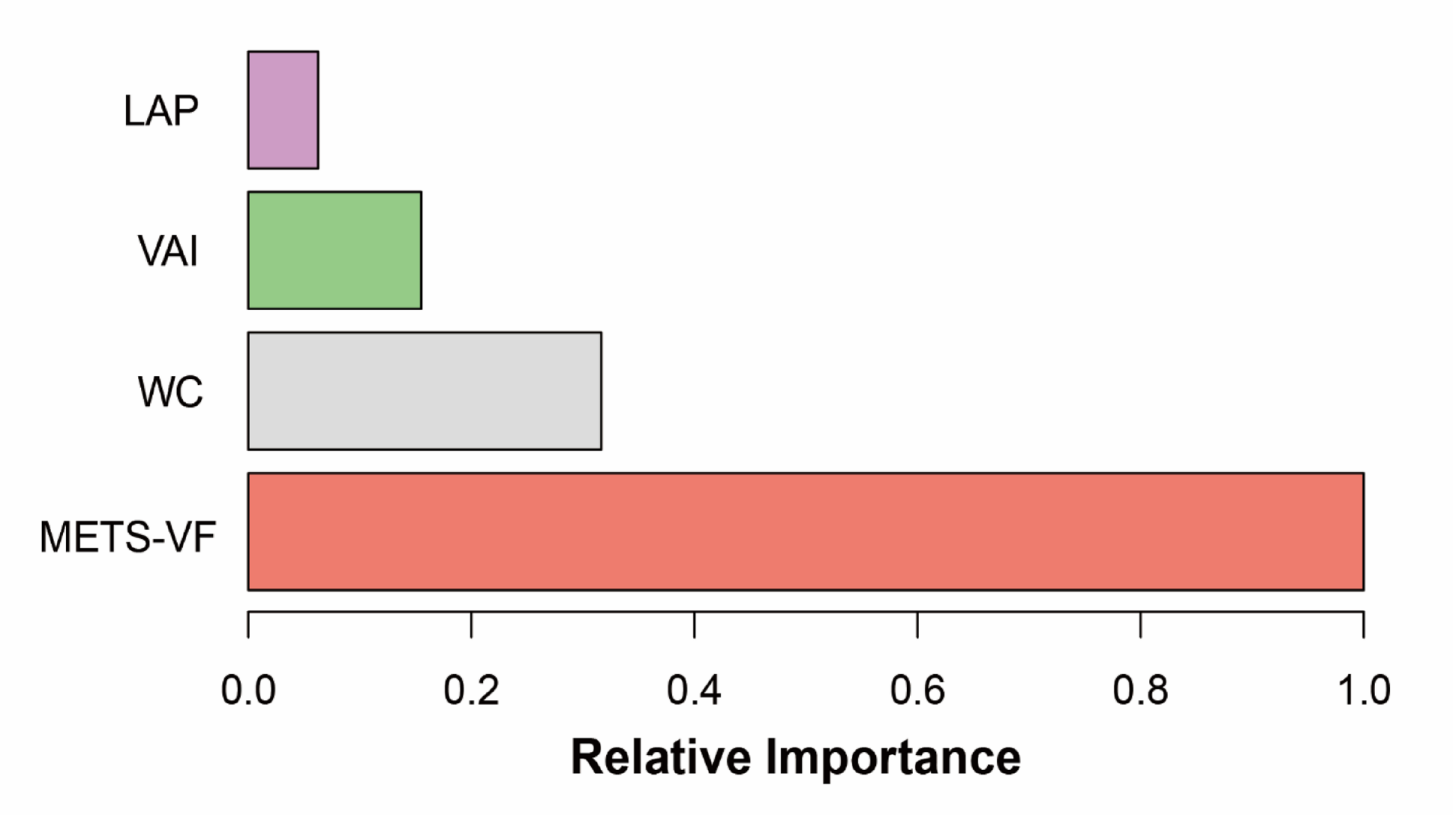
METS-VF ranked highest in XGBoost feature importance

**Fig. 6:**
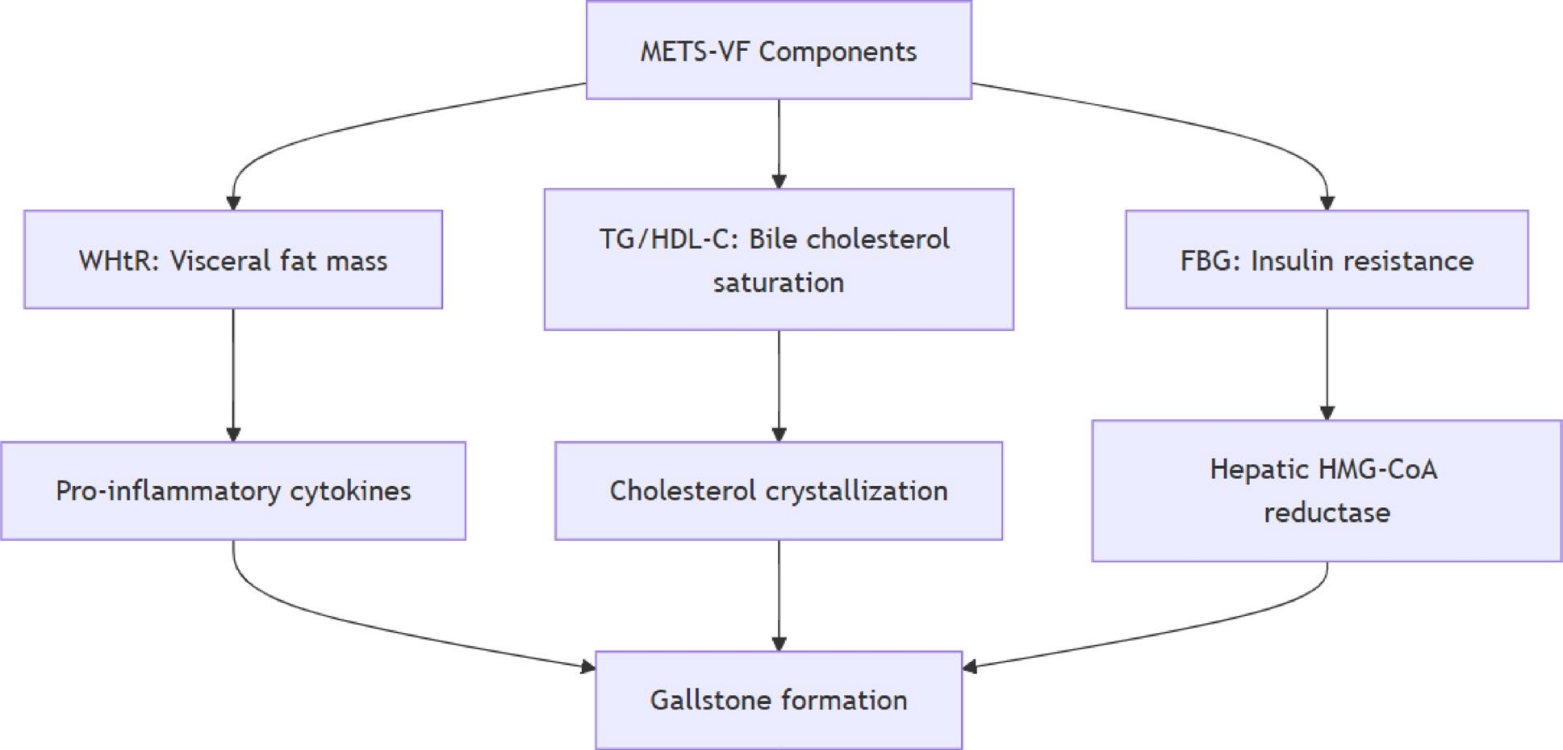
Association between METS-VF components (e.g., WHtR, TG, HDL-C) and gallstone pathogenesis

The XGBoost model, a foremost machine learning strategy, was engaged to establish the relative significance of several fat accumulation markers in the context of gallstones. METS-VF (visceral fat metabolic score) shows the highest predictive value for gallstone risk (relative importance close to 1.0), significantly outperforming other indicators:.WC(relative importance close to 0.2),VAI(relative importance close 0.4), LAP relative importance close 0.1. The results are detailed in Fig. 5. The machine learning evaluation ascertained that METS-VF is the leading marker of visceral fat accumulation related to gallstones, followed sequentially by WC, VAI, and LAP, and the Association between METS-VF components (e.g., WHtR, TG, HDL-C) and gallstone pathogenesis in Fig. 6.

## Discussion

To our awareness, this constitutes the initial cross-sectional study to probe the relationship between METS-VF and gallstone prevalence among U.S. adults. The findings revealed a significant association between METS-VF and the development of gallstones, which remained robust after adjusting for potential confounders.

Obesity is strongly associated with gallstones, especially in people with metabolic syndrome [3, 6]. Gallstones are common digestive issues, particularly prevalent among those with obesity and metabolic syndrome [7, 8]. She accuracy of METS-VF in measuring visceral fat has been validated by dual-energy X-ray absorptiometry (DXA), which demonstrates that METS-VF is more precise than BMI, WC, body weight, and WHtR [12]. This study demonstrated through DeLong’s test that the Metabolic Score for Visceral Fat (METS-VF) exhibits significantly superior predictive performance for gallstone risk compared to conventional obesity indices. As shown in Supplementary Table S1, METS-VF showed statistically significant differences when compared with waist circumference (Z = 4.12, *P* < 0.001), visceral adiposity index (Z = 6.85, *P* < 0.001), and lipid accumulation product (Z = 3.98, *P* < 0.001), with all comparisons maintaining significance after multiple testing correction. These findings extend previous research on the obesity-gallstone association [7] and establish, for the first time, METS-VF as the optimal body composition indicator for gallstone prediction. Moreover, a Turkish investigation has proven that METS-VF possesses the highest predictive efficacy for visceral fat assessment, outstripping LAP and VAI [13]. Since METS-VF is based on commonly used clinical indicators, it is a preferred method for assessing visceral fat oveveral studies have established a strong association between obesity and gallstones. For example, Stender et al. demonstrated a causal relationship between increased BMI and the risk of asymptomatic gallstones through Mendelian randomization, with a stronger effect in women [9]. Another recent study found a positive relationship between higher VAI and gallstone prevalence, which may also correlate with younger age at first gallstone surgery [10]. Other scholars have observed that a raised body roundness index (BRI) is linked with an increased gallstone risk among U.S. adults [11]. This current study provides a novel viewpoint by investigating METS-VF as an emerging marker of fat accumulation pertinent to gallstones. BMI is frequently used to assess obesity, but it does not distinguish between different types and distributions of fat, particularly visceral fat. Similarly, WC and WHtR cannot differentiate between visceral and subcutaneous fat. On the other hand, METS-VF provides a more precise reflection of metabolic health by considering factors like age, gender, WHtR, and METS-IR. Ter magnetic resonance imaging (MRI) and computed tomography (CT), which are more expensive and require specialized procedures. Therefore, using METS-VF in clinical practice can help identify high-risk individuals early and guide personalized prevention and intervention strategies. While METS-VF demonstrated statistically significant advantages over traditional adiposity indices in our analysis, we caution that its absolute predictive accuracy (AUC = 0.712) remains moderate for clinical decision-making. The relatively small AUC differences (e.g., 0.712 vs. 0.635 for WC) may not justify replacing conventional measures in practice without further validation. Future studies should assess whether combining METS-VF with other risk factors improves clinical utility.

The study highlights significant gender differences. AUC values for obesity-related indicators were higher in men than in women, suggesting that these indicators have a stronger predictive value for gallstone risk in males. This finding indicates that visceral fat accumulation in men may be more directly associated with gallstone risk, likely due to their distinct fat distribution and metabolic profiles. Men are more prone to accumulate visceral fat, particularly in the abdominal area, while women are more likely to store fat subcutaneously [14]. The superior predictive accuracy of METS-VF in males (AUC 0.681 vs. 0.679 in females) may reflect: The superior predictive accuracy of METS-VF in males (AUC 0.681 vs. 0.679 in females) may reflect:; Testosterone-driven visceral adipogenesis [10];Enhanced hepatic cholesterol uptake in males [19]; Sex-specific METS-VF thresholds; hormonal influences on fat distribution. This difference may account for the greater predictive accuracy of these indicators in men, as they more effectively capture visceral fat levels. Visceral fat serves as both an energy storage tissue and a regulator of systemic metabolism through the secretion of cytokines and hormones. Studies have demonstrated that visceral fat produces bioactive substances such as leptin, adiponectin, and resistin. These molecules play critical roles in regulating metabolism, modulating inflammatory responses, and influencing insulin sensitivity [15]. Leptin, for instance, controls appetite and energy balance while also affecting lipid metabolism and bile composition [16]. High leptin levels are strongly associated with cholesterol metabolism abnormalities, which can cause cholesterol supersaturation in bile and subsequently lead to cholesterol gallstone formation [17, 18]. Additionally, the inflammatory state of visceral fat tissue is considered one of the key factors in gallstone formation. Visceral fat is highly metabolically active and tends to induce chronic, low-grade inflammation [19]. In such inflammatory conditions, macrophages infiltrate the fat tissue, releasing pro-inflammatory factors like TNF-α and IL-6. These factors affect hepatic lipid metabolism and increase cholesterol levels in bile [20–22]. Chronic inflammation not only enhances the likelihood of cholesterol crystallization but also weakens gallbladder contraction, leading to bile stasis and promoting stone formation. Therefore, the METS-VF index reflects not only the extent of fat accumulation but also its metabolic activity and inflammatory state, all of which may play significant roles in the development of gallstones.

Our study demonstrates several strengths. We utilized a robust, representative sample, and applied weighted analysis to the data, which enhanced the reliability of our results. Additionally, we implemented a simplified scoring system, the METS-VF index, to examine the interaction between visceral fat and gallstone prevalence comprehensively. This method allowed us to better capture the metabolic complexities of visceral fat. However, certain shortcomings must be acknowledged. The cross-sectional format of this research identifies associations, yet it cannot establish causal relationships. Moreover, despite controlling for numerous confounding variables, residual confounding could remain.

Although this study utilized nationally representative NHANES data, several limitations regarding generalizability should be noted: First, the study population was restricted to U.S. adults, whose genetic backgrounds, dietary habits, and healthcare systems may differ significantly from other regions. For example, the visceral fat distribution patterns and high-carbohydrate diets common in Asian populations may affect the predictive performance of METS-VF. Second, the NHANES sampling methodology may introduce selection bias. Despite its complex survey design, certain subpopulations (e.g., homeless individuals) may be underrepresented.

These results provide key insights into the association between visceral fat and gallstones. While METS-VF demonstrates strong predictive value for gallstone prevalence in our cross-sectional analysis, the temporal relationship and potential causality require further investigation through longitudinal designs. The index may serve as a screening tool, but clinical monitoring decisions should integrate additional evidence. The components of METS-VF collectively reflect pathways implicated in gallstone pathogenesis. This multi-parametric design in Fig. 6 allows METS-VF to better represent the metabolic-inflammatory axis of gallstone formation than single anthropometric measures (e.g., WC or BMI). For example, in our cohort, METS-VF’s stronger association (OR = 2.02 in Model 3) may reflect its ability to simultaneously quantify fat distribution (WHtR), dyslipidemia (TG/HDL), and glucose metabolism (FBG) - all mechanistically linked to cholesterol crystallization [1,3,22].METS-VF is the core predictor of gallstone risk, with clinical applicability superior to traditional obesity indicators. METS-VF may enhance early gallstone screening in clinical practice. While logistic regression remains the gold standard for epidemiological association studies, XGBoost provided additional insights by quantifying the relative contribution of each adiposity index, independent of linear assumptions. The XGBoost analysis was exploratory, and its feature importance rankings should be validated in independent cohorts with standardized biomarker measurements.

Several limitations should be acknowledged. First, the cross-sectional design precludes causal inferences between METS-VF and gallstones. Reverse causality (e.g., gallstones affecting fat metabolism) or residual confounding cannot be ruled out.and Although we have adjusted for major known confounders, unmeasured variables such as dietary fiber intake, bile acid profiles and genetic factors may potentially influence the results. These findings require validation in prospective cohorts with repeated METS-VF measurements. Second, gallstone status was self-reported without imaging confirmation, which may lead to misclassification. And the NHANES design does not permit assessment of the temporal sequence between METS-VF measurement and gallstone diagnosis. As gallstone status was self-reported without imaging confirmation, we cannot determine whether Elevated METS-VF preceded gallstone formation (supporting predictive utility), or Existing gallstones influenced metabolic parameters (reverse causality).Future longitudinal studies with serial METS-VF measurements before gallstone development are needed to establish temporality。However, such misclassification is likely non-differential with respect to METS-VF, biasing effect estimates toward null hypotheses rather than inflating spurious associations. Future studies should validate these findings using imaging-confirmed gallstone diagnoses to improve accuracy. Third, our study could not differentiate between cholesterol and pigment gallstones or assess symptom status (asymptomatic vs. symptomatic), as NHANES relies on self-reported gallstone diagnosis without imaging or biochemical confirmation. Cholesterol gallstones, which account for 80% of cases in Western populations (Portincasa et al., Lancet 2006), are more strongly linked to metabolic disorders and visceral obesity. Future studies should incorporate imaging modalities (e.g., ultrasound or CT) to classify gallstone subtypes and evaluate their distinct associations with METS-VF. Fourth, our analysis could not adjust for certain lifestyle and pharmacological confounders, such as dietary composition (e.g., saturated fat or fiber intake), physical activity, or use of lipid-lowering medications (e.g., statins) and hormone therapies. These factors are known to modulate both visceral adiposity [7] and gallstone risk [5]. For instance, statins may reduce cholesterol saturation in bile, while hormone therapies can alter gallbladder motility. Although NHANES collects partial data on these variables, inconsistencies in measurement timing and completeness precluded their inclusion. This may introduce measurement error. Residual confounding from these unmeasured factors cannot be ruled out, and future studies should prioritize their systematic assessment.

## Conclusions

With data derived from a representative U.S. population sample, our investigation confirmed a strong association between METS-VF and gallstone prevalence (OR = 2.02, 95% CI:1.20–3.40),METS-VF showed relatively better predictive performance (AUC = 0.712, 95%CI:0.684–0.739) than WC, LAP and VAI (DeLong test *P* < 0.05), though all indices had modest discriminative ability (AUCs 0.530–0.712).

## Electronic supplementary material

Below is the link to the electronic supplementary material.

Supplementary Material 1

Supplementary Material 2

Supplementary Material 3

## Data Availability

The data used in this study are publically available in the NHANES database (www.cdc.gov/nchs/nhanes).
