# Supplementary figures and images for: METS-VF as a novel predictor of gallstones in U.S. adults: a cross-sectional analysis (NHANES 2017–2020)
Source: BMC Gastroenterol. 2025 Jul 31;25:547. doi: 10.1186/s12876-025-04161-x (PMC12315333; doi:10.1186/s12876-025-04161-x)

Supplementary Note 1


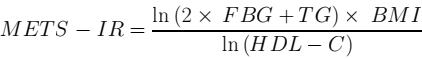


METS - VF = 4.466 +0.01 × (ln(METS - IR))3 +3.329 × (ln(WHtR))3 +0.319 × gender +0.594 × ln(age)

Supplement: Supplementary file 1 — Supplementary Material 1 [file 12876_2025_4161_MOESM1_ESM.docx]
