# Supplementary material for: METS-VF as a novel predictor of gallstones in U.S. adults: a cross-sectional analysis (NHANES 2017–2020)
Source: BMC Gastroenterol. 2025 Jul 31;25:547. doi: 10.1186/s12876-025-04161-x (PMC12315333; doi:10.1186/s12876-025-04161-x)
Supplement: Supplementary file 2 — Supplementary Material 2 [file 12876_2025_4161_MOESM2_ESM.docx]

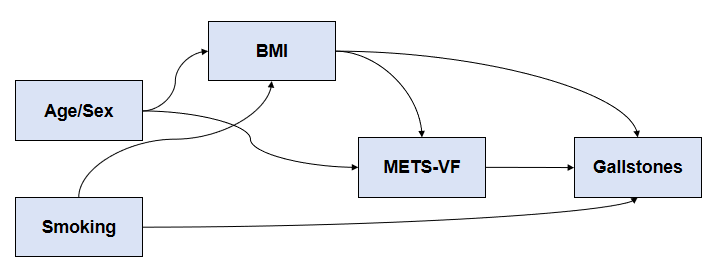


Supplementary Fig. 1 Directed acyclic graph (DAG) illustrating the hypothesized causal relationships between METS-VF, gallstones, and covariates in the NHANES 2017-2020 cohort.
