# Supplementary material for: METS-VF as a novel predictor of gallstones in U.S. adults: a cross-sectional analysis (NHANES 2017–2020)
Source: BMC Gastroenterol. 2025 Jul 31;25:547. doi: 10.1186/s12876-025-04161-x (PMC12315333; doi:10.1186/s12876-025-04161-x)
Supplement: Supplementary file 3 — Supplementary Material 3 [file 12876_2025_4161_MOESM3_ESM.docx]

Supplementary Table S1. DeLong Test for AUC Comparisons

| **Comparison** | **Z-score** | **p-value** | **Adjusted p-value*** |
| --- | --- | --- | --- |
| METS-VF vs WC | 4.12 | <0.001 | <0.001 |
| METS-VF vs VAI | 6.85 | <0.001 | <0.001 |
| METS-VF vs LAP | 3.98 | <0.001 | <0.001 |

*Bonferroni correction for multiple comparisons

abbreviations:

WC: Waist Circumference ；VAI: Visceral Adiposity Index；LAP: Lipid Accumulation Index；METS-VF: Metabolic Score for Visceral Fat.
